# Impact of the Russia-Ukraine conflict on the quality and quantity of Malaysia’s palm oil production: A time series analysis

**DOI:** 10.1371/journal.pone.0302405

**Published:** 2024-05-06

**Authors:** Azlizan Mat Enh, Hasrina Mustafa, Fahri Ahmed, Andika Wahab

**Affiliations:** 1 Research Center for History, Politics and International Affairs, Faculty of Humanities and Social Sciences, Universiti Kebangsaan Malaysia, Bangi, Malaysia; 2 School of Communication, Universiti Sains Malaysia, Gelugor, Malaysia; 3 Institute of Malaysian and International Studies, Universiti Kebangsaan Malaysia, Bangi, Malaysia; National Cheng Kung University, TAIWAN

## Abstract

This study investigates the effects of the Russia-Ukraine conflict on the quality and quantity of Malaysia’s palm oil production through a time series analysis. The study uses three primary factors to evaluate palm oil production: the Monthly Oil Extraction Rate (OER), the Monthly Fresh Fruit Bunch (FFB) Yield, and the Monthly Oil Exports. The results indicate that the Russia-Ukraine conflict significantly impacted the quality and quantity of palm oil production in Malaysia. Marginal declines in both the quality and quantity of palm oil produced at the onset of the conflict indicate a slight but significant decline in palm oil production during the next four-year period.

## Introduction

The Russia-Ukraine conflict, which commenced in February 2022, has had significant impact on the commodities market worldwide. As the conflict between Russia and Ukraine continued in 2023, a heightened level of global commodity price synchronisation and price fluctuations for such agricultural commodities as corn, wheat, barley, and sunflower oil resulted. Scholars [1] have already studied the influence of the Russia-Ukraine war on agricultural commodities. But the ongoing conflict also disrupted significantly the supply chain of such edible oils as sunflower oil, thus causing an important increase in demand for palm oil [2]. Consequently, the price of palm oil has escalated significantly. The events in question have had significant effects on Malaysia, a leading global producer of palm oil.

However, there is a shortage of comprehensive scholarly studies of the ramifications of the ongoing Russia-Ukraine conflict on both the quality and quantity of palm oil output in Malaysia [3, 4]. It should be noted that the quality and quantity of palm oil production are impacted by the quality of fertilisers used. For many years, Malaysia has relied heavily on Russia, the world’s leading producer of high-quality fertilisers such as potash, urea, and ammonia [5], for its supply of fertiliser. The ongoing Russia-Ukraine conflict has caused significant price hikes for fertilisers globally, which subsequently created food security issues at the national level. As fertilisers make up about 40% of the cost of palm oil production [6], the increase in the price of fertilisers and the interruptions in the supply of fertilisers have serious long-term impacts on Malaysia’s palm oil industry. Hence, it is imperative to understand the impact of the Russia-Ukraine conflict on Malaysia’s monthly oil extraction rate (OER), fresh fruit bunch (FFB) production, and oil exports between 2014 and 2023. Such a study would provide critical insights into how and whether palm oil production have been affected by the Russia-Ukraine conflict. In addition, since the conflict is ongoing, it is crucial to understand the magnitude of the impact the Russia-Ukraine war has on the quality and quantity of palm oil produced in Malaysia before and after the commencement of the war and its forecasted impact in the future.

A review of past literature indicated a number of research that explicitly focused on the impact of geopolitical events on palm oil. For example, scholars [7] claimed that uncertainty and crude oil prices have always had a complex relationship. Three types of uncertainty, i.e., economic policy uncertainty, geopolitical risk uncertainty and climate policy uncertainty, have exacerbated abnormal fluctuations in the energy market, making crude oil prices volatile more and more frequently, especially from the perspective of the financial attribute of crude oil. First, there are significant differences in the overall impact of the three types of uncertainties on crude oil prices, and this heterogeneity is reflected in quantiles of the peak impact intensity, the impact direction, and the fluctuation change. Second, the impact of the three types of uncertainties on crude oil prices differ significantly at different times Third, the impact directions and fluctuations of the three types of uncertainties on crude oil prices are heterogeneous at different times. Scholars [8] investigated the impact of geopolitical risk, U.S. economic policy uncertainty, financial stress and market volatility on U.S. and Brazilian ethanol and Malaysian palm oil prices. They found that uncertainty led to undesired price fluctuations in biofuel commodities. Moderate uncertainty only influenced prices moderately, while considerable uncertainty caused significant or extreme changes in ethanol and palm oil prices.

War, too, may affect palm oil prices. Scholars [9] claimed that the Russia–Ukraine war has various negative socioeconomic impacts that are now being felt internationally and may worsen global food security. Understanding how conflict-related disruptions in global food and fertiliser markets might affect price and availability is critical for understanding their overall impact on global food security. They argued that the halt of Ukrainian exports has led to significant constraints on the production of such agricultural products as fertilisers, which poses a significant threat to the quality and quantity of palm oil. It may reduce their use and crop yields. However, these claims are contradicted by a study in Colombia. Other scholar [10] studied the repercussions of the civil war and their effects on palm oil export. He found that the civil war inhibited economic globalisation, which arguably produces economic growth. He found that violence in Colombia’s civil war facilitated palm oil exports.

Some studies have focused on palm oil production and its internal and external shock sensitivity. Another researchers [11] investigated the impact of external and internal shocks on Malaysian palm oil (MPO) prices, using the Structural Vector Autoregressive Model (SVAR). The empirical result from the impulse–response function (IRF) shows that the shock in the Infectious Disease Volatility Tracker (IDVT) significantly impacted Malaysian palm oil prices, suggesting that the MPO is exposed to external factors. In addition, among the external variables tested, the IDVT shows the longest-lasting and highest positive impact on Malaysian palm oil prices. These results follow forecast error variance decomposition, indicating that IDVT shock can explain a huge portion of MPO prices, especially over extended periods. In addition, the same researchers [11] investigated the relative importance of external and internal shocks on the movement of palm oil prices in Malaysia. Using a structural vector autoregressive (SVAR) model on quarterly data from 1990 to 2019, the findings reveal that external shocks dominate the palm oil price. Various factors, including changes in crude oil prices, the cost of alternative items such as soybean oil, rapeseed oil, and sunflower oil, fluctuations in the global palm oil market, and variations in foreign income levels influence the short and medium-term fluctuations in the price of palm oil. The findings also suggest that an upsurge in the price of soybean oil has a more significant impact on the price of palm oil, compared to fluctuations in the prices of rapeseed oil or sunflower oil, respectively. Likewise, shocks to incomes in India and the Netherlands significantly impact the price of palm oil more than a shock to incomes in Malaysia’s other trading partners.

Malaysia is the second largest oil palm producer in the world after Indonesia. Together, Indonesia and Malaysia account for approximately 80 per cent of the world’s palm oil producers [12]. In 2020, Malaysia accounted for 25.8% and 34.3% of the world’s palm oil production and exports, respectively. India, China, Japan, Turkiye, Netherlands, Saudi Arabia Kenya, Philippines, Pakistan and Iran are the main importers of Malaysia’s palm oil products, accumulating a total of 9,809,271MT of palm oil products, which made up 62.4% of the country’s palm oil exports [13].

Malaysia’s palm oil industry has consistently recorded growth in not just the quantity of palm oil produced but also the quantity exported. To measure production or output performance, indicators or variables such as the Monthly Oil Extraction Rate (OER), Monthly Fresh Fruit Bunch (FFB), Yield of Oil Palm Estates and Monthly Oil Exports (in tonnes) are used. For many years, the OER, FFB and Monthly Oil Exports (in tonnes) have recorded consistent growth, except during the outbreak of COVID-19 from 2020–2022. To illustrate the situation, the average FFB yield in 2021 had declined to 15.47 tonnes per hectare, which was 7.5% lower than the average FFB yield in 2020. Similarly, crude palm oil production had plummeted by 5.4% year-on-year compared to crude palm oil production the previous year. The noticeable decrease in palm oil production during the pandemic was primarily due to the various travel restrictions and lockdowns imposed, which significantly affected productivity. The situation was further aggravated by the total international border closure which affected the hiring of foreign labourers [14, 15].

Amidst the various challenges brought about by the COVID-19 pandemic, Malaysia’s palm oil industry suffered another blow because of the Russia-Ukraine conflict which started in February 2022. The ongoing conflict has impacted Malaysia’s palm oil industry positively and negatively. On the positive side, the conflict has led to a significant price hike for Crude Palm Oil (CPO), from an average of RYM4,407 (approximately USD 947.74) per tonne in 2021 to RYM5,087 (approximately USD 1093.98) per tonne in 2022, due to the disruption in supply of sunflower oil from Russia and Ukraine [14]. On the negative side, the war between Russia and Ukraine disrupted the supply of fertilisers to Malaysia’s palm oil industry. As Malaysia is highly dependent on Russia for its fertiliser supply, a disruption in the fertiliser supply would affect the quantity and quality of palm oil production, which can be measured based on OER, FFB and Monthly Oil Export Rate. In the present study, these factors are selected as the key indicators for assessing the performance of the palm oil industry in Malaysia.

The Oil Extraction Rate (OER) is selected as a crucial indicator for assessing the quality of palm oil. The OER measures the effectiveness of the oil extraction procedure, as it provides insight into the percentage of oil that may be obtained from FFB. A higher OER indicates superior palm oil quality, as it signifies a greater yield of oil obtained from FFB. OER is subject to various elements, including the level of ripeness of FFB, the effectiveness of the milling procedure, and the overall quality of the palm oil fruits. Hence, the OER is a significant measure for assessing the quality of palm oil production in Malaysia [16]. The Monthly Fresh Fruit Bunch (FFB) Yield of Oil Palm Estates is also selected as a key indicator to assess the Malaysia’s palm oil performance because it is a significant indicator of the quantity of palm oil produced. FFB, specifically, the volume of FFB generated per unit area of land, often expressed in hectares, measures the efficiency and output of oil palm plantations. A higher FFB yield indicates increased productivity and a higher volume of palm oil production. The production of FFB is subject to various factors, including the age of the oil palm trees, soil fertility, climate conditions and agricultural practices. Hence, the FFB yield serves as a crucial factor for assessing palm oil output in Malaysia [17].

Lastly, the monthly oil exports, measured in tonnes, is also selected as one of the factors in this study because it serves as an indicator of the demand for Malaysian palm oil in the international market. This factor denotes the quantity of palm oil that is exported from Malaysia to different countries. An increased level of oil exports signifies an increased demand for Malaysian palm oil and an increased level of palm oil production. The exportation of oil is subject to various factors, including the global demand for palm oil, the competitiveness of Malaysian palm oil, and trade policies. Hence, palm oil output in Malaysia can be effectively gauged by examining the country’s oil exports [18]. In summary, the evaluation of palm oil production in Malaysia necessitates the consideration of OER, FFB yield, and Oil Exports. These factors are critical to understanding the efficiency of the oil extraction process, the productivity level of palm oil fields and demand for Malaysian palm oil in the global market. As such, this study assessed palm oil production based on these variables as they play a critical role in determining the quantity and quality of palm oil in Malaysia.

Previous studies have shown how the Russia-Ukraine conflict has affected the markets for various agricultural commodities. As Malaysia’s palm oil industry is the second largest in the world, it is of utmost importance to evaluate the extent to which the Russia-Ukraine war has impacted the quantity and quality of palm oil produced. The research objectives (ROs) of the study first assess whether there is any significant impact on the quality and quality of palm oil produced in Malaysia before (January 2014 to February 2022) and during the Russia-Ukraine war (March 2022 to December 2023) to clearly determine whether Malaysia’s palm oil industry has been significantly affected by the conflict. Second, it forecasts the next four years (January 2024 to December 2027) palm oil quantity and quality based on historical data to ascertain to what extent Russia-Ukraine’s war can affect Malaysia’s palm oil industry in the future. The forecasted data is yielded since the conflict is still ongoing and as such its effect on Malaysia’s palm industry is very likely.

The research objectives for the study are broken down as follows:

### RO1: To Assess the Impact of the Russia-Ukraine Conflict on the Quantity of Malaysian Palm Oil Production

This objective aims to determine how the Russia-Ukraine conflict has affected the quantity of palm oil production in Malaysia before and after the conflict began. The research bases the assessment on two prime factors: the Monthly FFB Yield of Oil Palm Estates, which represents the productivity of the oil palm estates, and the Monthly Palm Oil Export Rate, which reflects the demand for Malaysian palm oil in the global market.

### RO2: To Evaluate the Impact of the Russia-Ukraine Conflict on the Quality of Malaysian Palm Oil Production

This objective aims to understand how the Russia-Ukraine conflict has influenced the quality of palm oil production in Malaysia and whether there is any significant impact on Malaysia’s palm oil quality before and after the Russia-Ukraine conflict. The evaluation is performed using the Monthly OER, which is a significant factor in determining palm oil quality and indicates the efficiency of the oil extraction process.

### RO3: To Forecast the Quantity and Quality of Malaysian Palm Oil Production for the Next Four Years

This objective aims to forecast the future trends in the quantity and quality of palm oil production in Malaysia over the next four years from January 2024 to December 2027. The forecast is based on the historical monthly data of FFB, OER and Oil Export of palm oil. The forecast analyses the monthly patterns of these data from 2014 to 2023. It takes into account the potential ongoing effects of the Russia-Ukraine conflict on the palm oil industry in Malaysia to derive its output.

## Method and materials

Monthly data from 2014 to 2023 was used in this research. The data set was subjected to time series analysis to identify patterns, trends, and relationships to predict future values. Time series analysis, a statistical technique that deals with time-ordered data points, is widely used in such fields as economics, finance, and engineering [19]. One of the most common models used in time series analysis is exponential smoothing, which assigns exponentially decreasing weights to past observations [20]. The exponential smoothing model is particularly efficient in analysing data with trends and seasonal fluctuations which align well with the characteristics of time series data from the palm oil industry, which exhibits such patterns because of market dynamics and seasonal agricultural cycles. Secondly, the model’s proven track record in economic contexts, as shown in the study by a team of researchers [21] on Portuguese e-commerce retail sales, indicates its applicability in analysing industry-related time series data. As this model is adept at capturing complex patterns within economic data, it is a robust choice for achieving the research objectives of accurately forecasting and understanding the impact of geopolitical events. In our case, it is particularly appropriate to use the exponential smoothing model to analyse data to evaluate the impact of the Russia-Ukraine conflict on the quantity and quality of palm oil produced by Malaysia.

However, there are three types of Exponential Smoothing models used in time series analysis. Which one is used depends on specific characteristics of the data. Simple Exponential Smoothing (SES), which applies a single smoothing factor, is best suited for time series without a trend or seasonal pattern. Holt’s Linear Exponential Smoothing, an extension of SES, is designed to analyse data with a trend but no seasonality. It introduces an additional parameter to capture the trend component [20]. Holt-Winters’ Exponential Smoothing, also known as Triple Exponential Smoothing, is used to analyse data with both trend and seasonality, as it involves three smoothing parameters for the level, trend, and seasonal components [22]. A suitable exponential smoothing method is required to interpret this study’s data well. Hence, an expert modeler was chosen to forecast future values based on the identified patterns in the time series analysis. To analyse the data effectively, the expert modeler must evaluate the different exponential smoothing categories to identify the most suitable model for the dataset. In our case, the expert modeler selected the simple exponential smoothing model [20].

Simple Exponential Smoothing is a time series forecasting model that is particularly suitable for data with no clear trend or seasonality [23]. It uses a single smoothing parameter, α (alpha), which determines the weight assigned to the most recent observation. The value of α ranges from 0 to 1, with higher values giving more weight to recent observations and lower values giving more weight to historical observations [20]. The choice of simple exponential smoothing for this research is justified by the nature of the data set and the results of the expert modeler. The data set does not exhibit clear trends or seasonality, making simple exponential smoothing an appropriate choice. Additionally, the expert modeler’s evaluation confirmed that simple exponential smoothing provides the best fit for the data set.

To assess the accuracy and precision of the Simple Exponential Smoothing model, several evaluation metrics were used, including Mean Absolute Error (MAE), Root Mean Square Error (RMSE), Mean Absolute Percentage Error (MAPE), R-Square, and P-Values. **Mean Absolute Error (MAE):** MAE measures the average absolute difference between observed and predicted values. It provides an indication of the overall accuracy of the model and is particularly useful for assessing the magnitude of the errors [24]. By understanding the average magnitude of errors, researchers can gauge how well the model performs in predicting palm oil production values. **Root Mean Square Error (RMSE):** RMSE is the square root of the average of the squared differences between the observed and predicted values. It is a commonly used metric for assessing the accuracy of a model and is sensitive to large errors [25]. In the context of this study, where precise predictions are vital, understanding the impact of large errors is crucial for refining the model. **Mean Absolute Percentage Error (MAPE):** MAPE measures the average absolute percentage difference between observed and predicted values. It is a useful metric for assessing the relative accuracy of a model, especially when comparing models with different scales [26]. Given the different scales of data in palm oil production over the different periods studied, MAPE helps researchers understand the model’s performance in relative terms. **R-Square:** R-Square, also known as the coefficient of determination, measures the proportion of the variance in the dependent variable that is explained by the independent variables in the model. It indicates how well the model fits and is useful for assessing the explanatory power of the model [27]. **P-Values:** P-Values are used to assess the statistical significance of the model parameters. A low P-Value (typically less than 0.05) indicates that the parameter is statistically significant at a 95% confidence level and contributes to the explanatory power of the model [28]. The assessment metrics are used to evaluate the performance of the Simple Exponential Smoothing model on the data set.

## Results

The analysis is conducted using three different factors of palm oil production: quality based on OER (in percentage) andquantity based on FFB (in tonnes per hectare), and quantity based on Oil Export Rate (in tonnes). Table 1 shows the assessment metrics used to evaluate the performance of the Simple Exponential Smoothing model on the data set.

**Table 1:** MAE, RMSE, MAPE, R-Square, stationary R-Square values and P-Value.

Palm Oil	RMSE	MAE	MAPE	R-Square	P-Value
Quality Based on Extraction Rate (in percentage)	0.246	0.190	0.949	0.646	0.005
Quantity Based on Fresh Fruit Bunch (in tonnes per hectare)	0.136	0.108	7.917	0.586	0.000
Palm Oil Quantity Based on Oil Export Rate (in tonnes)	189319.486	144961.723	10.759	0.540	0.004

Table 1 shows that the quality of palm oil production, as measured by the OER in percentage, exhibited a relatively low RMSE of 0.246 and MAE of 0.190, indicating that the model’s predictions are close to the observed values. The R-Square value of 0.646 indicates that approximately 64.6% of the variance in the quality of palm oil, as measured by the OER, is explained by the model. The remaining 35.4% of the variance might be attributed to factors not included in the model, such as environmental conditions, variations in farming practices, or other external factors. The MAPE of 0.949 suggests that the model’s predictions are, on average, off by 0.949, indicating the highly accurate predictions for the quality of palm oil based on OER. The P-Value tested for OER is 0.005, which is lower than 0.05, indicating that the model parameters are statistically significant. In other words, it means that the patterns of monthly OER analysed from 2014 to 2023 and predicted from 2024 to 2027 are significant.

As per Table 1, the quantity of palm oil production, as measured by FFB in tonnes per hectare, shows a low RMSE of 0.136 and MAE of 0.108, indicating accurate predictions. The R-Square value of 0.586 indicates that approximately 58.6% of the variance in the quantity of palm oil, as measured by FFB, is explained by the model. The unexplained 41.4% could be due to such factors as differences in soil quality, amount of rainfall, or agronomic practices not accounted for in the model. The MAPE of 7.917 suggests that the model’s predictions are, on average, off by 7.917, indicating a moderate level of accuracy in predicting the quantity of palm oil based on FFB. The P-Value tested for FFB results in 0.000, which is lower than 0.05, indicating that the model parameters are statistically significant. In other words, the patterns of monthly FFB analysed from 2014 to 2023 and predicted from 2024 to 2027 are significant.

Lastly, the palm oil quantity based on the Oil Export Rate in tonnes exhibited in Table 1 shows a higher RMSE of 189319.486 and MAE of 144961.723 compared to the other two aspects, indicating slightly larger errors in the predictions. The R-Square value of 0.540 suggests that about 54% of the variance in the palm oil quantity based on the Oil Export Rate is explained by the model. The remaining 46% that is unexplained could be due to external market factors, global demand and supply dynamics, or geopolitical events that influence export rates. The MAPE of 10.759 indicates that the model’s predictions are, on average, off by 10.759, suggesting that there is room for improvement in its predictive accuracy. The P-Value tested for Oil Export Rate results in 0.004, which is lower than 0.05, indicating that the model parameters are statistically significant. In other words, it means that the patterns of monthly Oil Export Rate analysed from 2014 to 2023 and predicted from 2024 to 2027 are significant.

The assessment metrics results derived in Table 1 indicate that the model provides a good fit for the data and produces accurate and precise predictions. The relatively low values of MAE, RMSE, and MAPE indicate that the model’s predictions are close to the observed values. The high R-Square value indicates that the model explains a large proportion of the variance in the data. The low P-Values indicate that the model parameters are statistically significant. In conclusion, the simple exponential smoothing model is identified as the most suitable time series model for this research based on the characteristics of the data set, evaluations by the expert modeler, and the assessment metrics. The model is used to analyse data from 2014 to 2023 and predict the next four years of data from 2024 to 2027.

**Fig 1:**
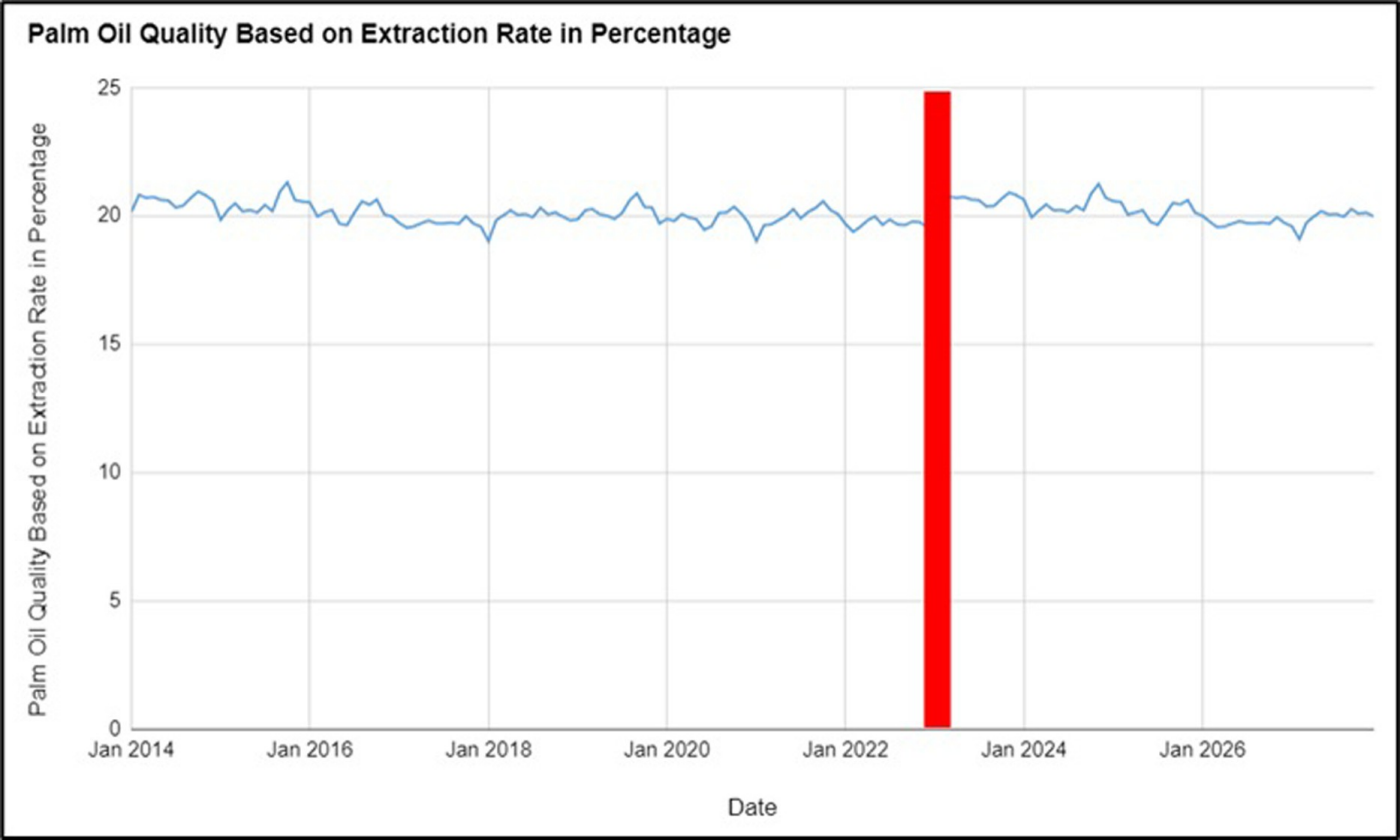
Palm oil quality based on Oil Extraction Rate (OER) in percentage observed values from 2014–2023 and forecasted values from 2024–2027.

The next sets of figures are the results of OER, FFB and Oil Export Rate from January 2014 to December 2027, pertaining to the research objectives of first evaluating the impact of the Russia-Ukraine conflict on the quality and quantity of palm oil produced in Malaysia before (January 2014 to February 2022) and after the Russia-Ukraine conflict (March 2022 to December 2023), and second, the forecasted quantity and quality of palm oil produced in Malaysia for the next four years (January 2024 to December 2027). The Tables show the forecasted values of the factors for the next four years from January 2024 to December 2027. The results derived from Fig 1 represent the Monthly Oil Extraction Rate (OER) in Malaysia from January 2014 to December 2027. OER measures the quality of palm oil, with higher values indicating better quality. An analysis of the data shows variations in the quality of palm oil before (January 2014 to February 2022) and during the Russia-Ukraine conflict (March 2022 to December 2023). Table 2 represents the consecutive effects on the forecasted values from 2024 to 2027.

**Table 2:** Forecasted values for palm oil quality based on Oil Extraction Rate (OER) in percentage 2024–2027.

Month / Year	2024	2025	2026	2027
Jan	20.63	20.58	20.00	19.60
Feb	19.95	20.54	19.76	19.09
Mar	20.21	20.05	19.57	19.74
Apr	20.45	20.14	19.59	19.98
May	20.22	20.22	19.70	20.19
Jun	20.23	19.77	19.80	20.06
Jul	20.15	19.66	19.72	20.07
Aug	20.39	20.07	19.71	19.97
Sep	20.22	20.51	19.74	20.27
Oct	20.86	20.45	19.70	20.09
Nov	21.25	20.62	19.95	20.13
Dec	20.70	20.13	19.73	19.99

**Fig 2:**
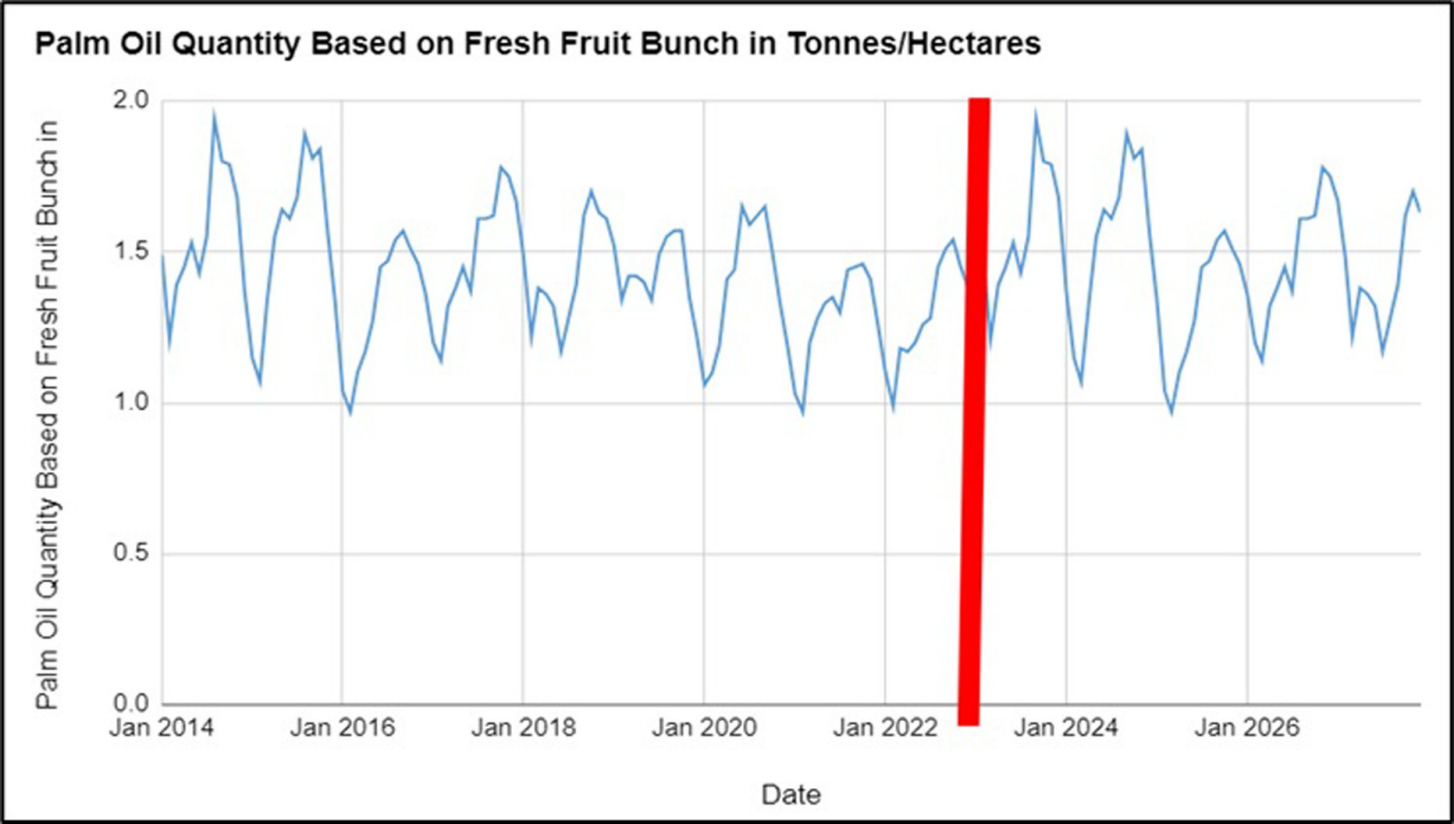
Palm oil quantity based on Fresh Fruit Bunch (FFB) in tonnes/hectares observed values from 2014–2023 and forecasted values from 2024–2027.

The data from Fig 2 represent the Monthly FFB in Malaysia from January 2014 to December 2027. FFB measures quantity of palm oil, with higher values indicating higher quantity. An analysis of the data shows the variations in the quantity of palm oil produced in Malaysia before (January 2014 to February 2022) and after the Russia-Ukraine conflict (March 2022 to December 2023). Table 3 represents the consecutive effects on the forecasted values from 2024 to 2027.

**Table 3:** Forecasted values for palm oil quantity based on Fresh Fruit Bunch (FFB) in tonnes/hectares from 2024–2027.

Month / Year	2024	2025	2026	2027
Jan	1.37	1.34	1.36	1.67
Feb	1.15	1.04	1.20	1.48
Mar	1.07	0.97	1.14	1.22
Apr	1.33	1.10	1.32	1.38
May	1.55	1.17	1.38	1.36
Jun	1.64	1.27	1.45	1.32
Jul	1.61	1.45	1.37	1.17
Aug	1.68	1.47	1.61	1.28
Sep	1.89	1.54	1.61	1.39
Oct	1.81	1.57	1.62	1.62
Nov	1.84	1.51	1.78	1.70
Dec	1.57	1.46	1.75	1.63

**Fig 3:**
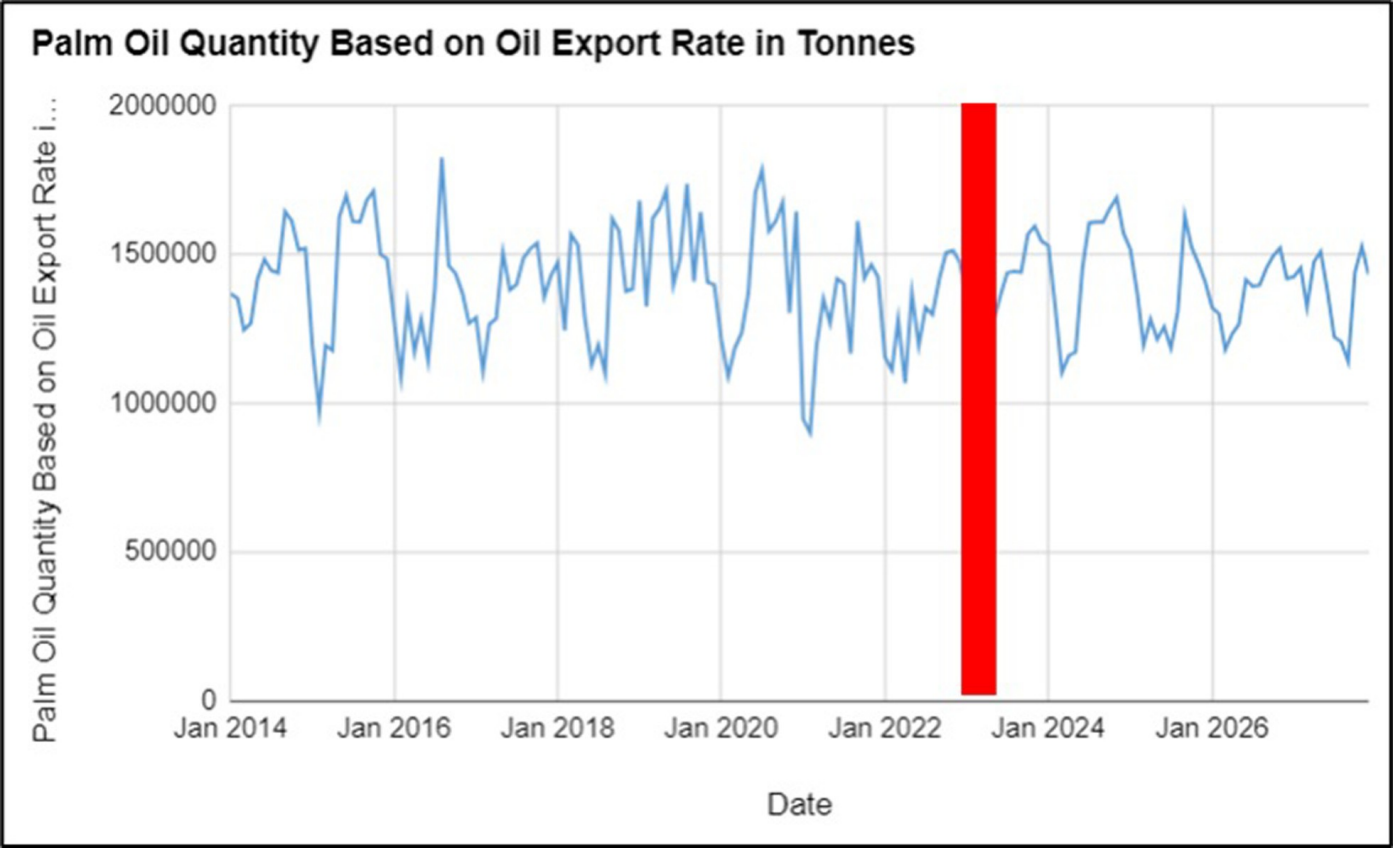
Palm oil quantity based on Oil Export Rate (in tonnes) observed values from 2014–2023 and forecasted values from 2024–2027.

The data from Fig 3 represents the Monthly Export Rate in Malaysia from January 2014 to December 2027. Export Rate is also a measure of the quantity of palm oil produced, with higher values indicating higher quantity. The data indicate that the quantity of palm oil produced varied before (January 2014 to February 2022) and after the Russia-Ukraine conflict (March 2022 to December 2023). Table 4 represents the consecutive effects on the forecasted values from 2024 to 2027.

**Table 4:** Forecasted values for palm oil quantity based on Oil Export Rate (in tonnes) from 2024–2027.

Month / Year	2024	2025	2026	2027
Jan	1528995	1517228	1320848	1424170
Feb	1319211	1369143	1300278	1454339
Mar	1103613	1194782	1180518	1323252
Apr	1159986	1282592	1233572	1474379
May	1171082	1214362	1265018	1509233
Jun	1453832	1256721	1415023	1373861
Jul	1605212	1185249	1393165	1221855
Aug	1608130	1309340	1396862	1206189
Sep	1608237	1629781	1453690	1140064
Oct	1653430	1524890	1494496	1438310
Nov	1689819	1469635	1521591	1525518
Dec	1571582	1407908	1418812	1432307

## Discussion

Between January 2014 and February 2022, before the Russia-Ukraine War began, the OER fluctuated between 19.01% and 21.31%. The average OER during this period was approximately 20.16%. Between March 2022 and December 2023, after the war began and continued, the OER ranged from 19.55% to 20.91%, with an average of approximately 20.23%, indicating a slight increase in the quality of palm oil produced. It has been forecasted that the OER between January 2024 and December 2027 will range from 19.09% to 21.25%, with an average OER for this period of approximately 20.17%. The forecasted average decrease in OER indicates that the quality of palm oil is likely to drop slightly over the next four years. However, the OER is not expected to deviate much from the range observed before the war. This slight decrease in OER in the forecasted values from January 2024 to December 2027 may be attributed to the supply chain disruptions caused by the conflict, as Ukraine is a major supplier of fertilisers used in palm oil production [29].

The data from January 2014 to February 2022 show fluctuations in the quantity of palm oil produced, based on FFB in tonnes/hectares. There is a seasonal pattern to the quantity of palm oil produced, with peaks usually occurring around August and September yearly. The average quantity of palm oil produced, based on FFB, during this period is approximately 1.41 tonnes/hectares. After the Russia-Ukraine war started in February 2022, the quantity of palm oil produced, based on FFB, decreased slightly, with the lowest value of 0.99 tonnes/hectares recorded in February 2022. The quantity of palm oil produced recovered in March 2022 and went as high as 1.94 tonnes/hectares. The average quantity of palm oil produced, based on FFB, from March 2022 to December 2023 was approximately 1.46 tonnes/hectares. The average forecasted quantity of palm oil produced based on FFB, in this period was approximately 1.44 tonnes/hectares. The slight increase in the average quantity of palm oil produced, based on FFB, after the war began might be because the war had not affected the production of palm oil. However, the slight dip in the average FFB after the conflict (1.46 tonnes/hectares) and the average forecasted FFB from January 2024 to December 2027 of 1.44 tonnes/hectares may be attributed to likely supply chain disruptions caused by the conflict in the future, as Russia is a major supplier of fertilisers used in palm oil production. In conclusion, the quantity of palm oil will be affected by the Russia-Ukraine conflict, leading to a slight decrease in palm oil production in the future years.

The data also show that the quantity of palm oil produced, based on the Oil Export Rate in tonnes, fluctuated from 2014 to 2023. The quantity of palm oil exports generally increased, with some fluctuations, from 2014 to 2019. In 2020, there was a noticeable decrease in the quantity of palm oil exports, which could be attributed to the global impact of the COVID-19 pandemic on trade and transportation. In 2021, while still lower than pre-pandemic levels, the quantity of palm oil exports showed a slight recovery. In 2022, the quantity of palm oil exports decreased further, especially after the Russia-Ukraine war started in February 2022. This could be due to supply chain disruptions and geopolitical tensions affecting the global trade of palm oil. Overall, before the Russia-Ukraine conflict (January 2014 to February 2022), the average Oil Export Rate was 1,409,233 tonnes. However, after the conflict (March 2022 to December 2023), the Oil Export Rate suffered a drastic drop of an average 1,384,792 tonnes. It shows that the conflict significantly impacted the quantity of palm oil. The forecasted values from January 2024 to December 2027 show a slight increase in the quantity of palm oil exports, with an average value of 1,390,669 tonnes. Although the forecasted values are better than the post-conflict’s average oil export rate of 1,384,792 tonnes, it is still lower than the average Oil Export Rate before the conflict (1,409,233 tonnes.) As such, future improvement will be slight because of supply chain disruptions, effects of the war on economy and trade, geopolitical tensions and the changes in demand, production and government policies [4, 30, 31].

When OER and FFB are analysed, a similar pattern is evident. Both factors show a slight increase after the Russia-Ukraine conflict (March 2022 to December 2023) but the increase does not continue in the future (January 2024 to December 2027). The slight drops in the average forecasted values of OER and FFB indicate that the quality of palm oil based on OER and quantity based on FFB will eventually catch up, causing a slight decline in both quantity and quality in future years. Furthermore, the Oil Export Rate’s average value shows it is already experiencing a drop after the Russia-Ukraine conflict (March 2022 to December 2023). Although a slight improvement is forecasted from January 2024 to December 2027, it is still lower than the average value before the conflict (January 2014 to February 2022), indicating that the demand for Malaysian palm oil in the international market will suffer because of the ongoing war.

## Conclusion

This study, which is supported by empirical data and broader economic analyses, concludes that the Russia-Ukraine conflict has significantly impacted the quality and quantity of palm oil produced by Malaysia. The observed decline in palm oil production aligns with reports from the Malaysian Palm Oil Council 2021 [32], which highlight such challenges as fertiliser shortages and labour supply constraints since mid-2021. Moreover, the conflict’s indirect effects on global food commodities and energy markets, in which Russia and Ukraine are key players, underscore the interconnectedness of global supply chains and their susceptibility to geopolitical events. The forecasting analysis, utilising a simple exponential smoothing model, indicates a sustained negative impact on palm oil production through 2027. This projection is crucial for stakeholders in the palm oil industry, as it signals the need for strategic adjustments and resilience-enhancing measures. However, while the model effectively captures a significant portion of the data’s variance and its parameters show statistical significance, as evidenced by the P-values, it is important to recognise its limitations. The model does not account for several external factors that might influence palm oil production, such as global market dynamics, environmental changes, and policy shifts. These factors, as indicated by a number of scholars [17, 33], play a critical role in shaping agricultural outputs and market behaviours. In summary, the study highlights the substantial, though not drastic, decline in Malaysia’s palm oil production that can be expected in the coming years, attributable to the ongoing Russia-Ukraine conflict and its cascading effects on the supply chain, economy, and trade. Future research should aim to incorporate additional variables and use more complex models to capture these external influences more comprehensively, thus enhancing predictive accuracy and providing a more holistic understanding of the palm oil industry’s future trajectory.

## Supporting information

S1 Dataset (XLSX)
